# Intelligence, Correspondence, &c.

**Published:** 1825-04-01

**Authors:** 


### INTELLIGENCE, CORRESPONDENCE, &c.

A General Infirmary, or rather an Hospital, is about to be erected in Chichester, on a large scale, and chiefly, we believe, through the active exertions of that able physician, Dr. John Forbes, now so favourably known to the profession. We have the Prospectus before us, and the Resolutions agreed to at a Meeting held on the 29th December last, His Grace the Duke of Richmond in the chair. We were gratified to see the following resolution among others :—" Resolved, that the thanks of this meeting be given to Dr. Forbes, for the zeal he has shewn in endeavouring to promote the desirable object for which this meeting is assembled; and for his exertions in procuring statements and plans of nearly every hospital in England." Sincerely do we wish success to the undertaking, and confidently do we anticipate an accession of valuable information for the profession at large, from the field which this institution will open for the talents of the physician abovementioned.

---

### *Factitious Mineral Waters at Brighton.*

In a late number, we noticed the new Institution at Brighton, under the direction of the celebrated Dr. Struve, of Dresden, for the preparation of factitious Mineral Waters of repute on the Continent. We are happy in affording some farther information relative to this undertaking, in the present quarter.

At the Institution, all preparations are now brought to a conclusion; but as Dr. Struve, who considers it incumbent on him to be present at the opening, cannot be in England before the middle of next month, the opening is finally fixed for the *first of May*.

The Pump-Room, upon which no expense has been spared, is situated at the back of the East Cliff, in a valley, formerly composing part of the Downs, but which, from its sheltered position, was converted into pleasure grounds, which have been laid out with great taste, and bid fair to become a favourite promenade with genteel company. The liveliness of the scene will undoubtedly be no less favourable than the security from the south west winds, or the use of mineral waters.

The waters which will be found at the Pump-Room this year will be the following, viz; *Warm Waters*—The Sprudel, Neubrunnen, Mühlbrunnen, and Theresicubrunnen of Carlsbad: the Krœncheu and Kesselbrunnen of Ems. *Cold Waters*—The Kreutzbrunnen and Ferdinandsbrunnen of Marienbad and Auschowitz: the Franzensbrunnen of Eger: the Pouhon of Spa: the Pyrmont and Seltzer Waters.

Some of the Factitious Mineral Waters of Carlsbad have lately been analyzed by Mr. Faraday of the Royal Institution. He will shortly publish a report of the results which are uncommonly favourable.

---

### *Literary Notice.*

Dr. Gordon Smith is preparing a Systematic Work on Medical Police.

The communication from Mr. COLVAN is received, and will be properly disposed of.

---

Mr. JESTON's Analysis of Opium came just in time to be acknowledged in this sheet.

---

The "Hypocritical Petition" from the medical men of Bath, against "Cruelty to Animals," is received.

☞ We consider it a disgrace to the Profession to address the Senate or the Public on any such subjects. It is downright hypocrisy, and we shall not fail to stigmatize it as such.

---

Mr. BATTLEY has pursued his Analysis of the Constituents of Opium to a considerable extent, but the intricacy of the subject will not allow him to lay the results before the Professional Public, before the next, or a succeeding No. of this Journal.

*N.B.* Early in May, Mr. BATTLEY means to throw open his Specimens of Materia Medica, his Pharmaceutical Preparations, and his Laboratory in general, for the inspection of the Professional Public.

---

*Sand Rock Spring, Isle of Wight.*

Mr. DUNCOMBE, 199, Fleet Street, is appointed Agent for the sale of the residual Salts and Waters alluded to at page 555 of this Number.

---

### OBITUARY.

Lately died, at his residence, in Sloane Street, Dr. Chisholm, a most meritorious Physician, whose writings are well known to every member of the profession. He fell a victim to disease of the prostate gland, for which he had the able advice and constant assistance of his friend, Mr. Brodie.

Dr. C. went to America, as Surgeon of a Highland Regiment, in the year 1774, in which regiment, the since celebrated Dr. Jackson was then assistant surgeon. For some reason unknown to us, Dr. Chisholm left that line of the Army, and went into the Artillery, in the year 1782. In 1794, he obtained the appointment of Inspector-General of Ordnance Hospitals in the West Indies, and, about that period, we believe, published the first edition of his Work on the Fever of Grenada. His writings and controversies respecting the Bulam fever, are well known, and although Dr. Bancroft was supposed to have the better of the controversy at the time, yet many subsequent and recent events have tended to strengthen the opinions of Dr. Chisholm, and weaken the arguments of his opponent. His papers in the Edinburgh Journal, especially those on fish-poison, the dracunculus, &c. have all proved the author to be a man of science, accurate observation, ingenuity, and professional zeal. During his residence as a physician at Clifton, we have reason to believe he was highly respected by his brethren, and confided in by the public at large. A few years ago, Dr. Chisholm gave up the duties of his profession, and travelled and sojourned in France, Switzerland, and Italy, for the sake of his children's education, and his own health. He returned to this country about a year ago, and we had hopes that he would have enjoyed the "age of ease," after "a youth of labour;" but fate ordained it otherwise! We met him only a few months ago, at a public medical dinner, and he seemed in the possession of very good health and spirits—we conclude, therefore, that some acute inflammation must have supervened on a chronic disease, which is seldom suddenly fatal. To sum up his character in three words—he was an able physician—a man of probity—an affectionate husband and parent. Who would desire a better epitaph?

